# Dendrite formation in Li-metal anodes: an atomistic molecular dynamics study

**DOI:** 10.1039/c9ra05067a

**Published:** 2019-09-04

**Authors:** Luis A. Selis, Jorge M. Seminario

**Affiliations:** Department of Chemical Engineering, Department of Electrical and Computer Engineering, Department of Materials Science and Engineering, Texas A&M University College Station TX 77843 USA seminario@tamu.edu +1-979-845-3301

## Abstract

Lithium-metal is a desired material for anodes of Li-ion and beyond Li-ion batteries because of its large theoretical specific capacity of 3860 mA h g^−1^ (the highest known so far), low density, and extremely low potential. Unfortunately, there are several problems that restrict the practical application of lithium-metal anodes, such as the formation of dendrites and reactivity with electrolytes. We present here a study of lithium dendrite formation on a Li-metal anode covered by a cracked solid electrolyte interface (SEI) of LiF in contact with a typical liquid electrolyte composed of 1 M LiPF_6_ salt solvated in ethylene carbonate. The study uses classical molecular dynamics on a model nanobattery. We tested three ways to charge the nanobattery: (1) constant current at a rate of one Li^+^ per 0.4 ps, (2) pulse train 10 Li^+^ per 4 ps, and (3) constant number ions in the electrolyte: one Li^+^ enters the electrolyte from the cathode as one Li^+^ exits the electrolyte to the anode. We found that although the SEI does not interfere with the lithiation, the mere presence of a crack in the SEI boosts and guides dendrite formation at temperatures between 325 K and 410.7 K at any C-rate, being more favorable at 325 K than at 410.7 K. On the other hand, we find that a higher C-rate (2.2C) favors the lithium dendrite formation compared to a lower C-rate (1.6C). Thus the battery could store more energy in a safe way at a lower C-rate.

## Abstract 2

Lithium dendrites (blue) growing through the cracks of a SEI (orange) covering a Li-metal anode (blue) while lithiation by Li-ions (purple). The electrolyte is not shown.

## Graphical abstract

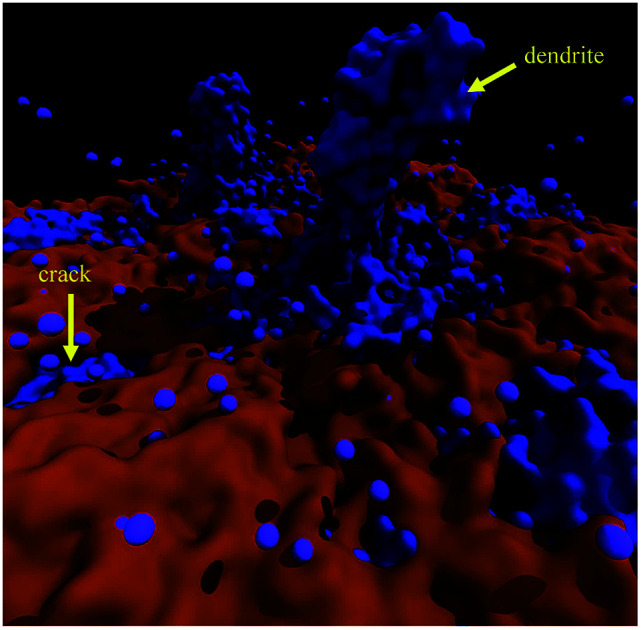

## Introduction

Combustion of fossil fuels is currently the principal source of energy for transportation and electric power needs. However, due to air pollution and limited reserves, renewable energy sources are of immediate interest. Lithium-ion batteries (LIB) are excellent devices to store energy and are now used in electric vehicles and electronic devices.^1,2^ With the growing demand for batteries with higher energy density, cathodes and anodes with greater theoretical capacity for the next generation of LIB are needed.^2–5^

Lithium-metal is an ideal anode for rechargeable LIBs due to its low density 0.59 g cm^−3^,^6^ very low absolute electrode potential of 1.40 V (−3.04 V *vs.* standard hydrogen electrode)^7–9^ and its extremely high theoretical specific capacity of 3860 mA h g^−1^ (ref. 9–11) compared with current commercial graphite anodes with densities between 2.09 and 2.23 g cm^−3^ and absolute electrode potential of 1.52 V (+0.12 V *vs.* Li/Li^+^)^12^ and a theoretical specific capacity of 372 mA h g^−1^.^13,14^ However, problems such as Li dendrite growth and cracking have limited the practical application of lithium-metal batteries;^15–18^ lithium dendrite growth can expedite the capacity fade of a battery and make batteries vulnerable to security issues such as burning or destruction of devices during the charge and discharge cycles with liquid electrolytes.^8,19,20^

Lithium dendrite growth has been extensively investigated in the last decades,^21^ but lithium dendrite growth is still almost inevitable during charge and discharge cycles of the battery.^22,23^ Lithium dendrites growth has been detected at the solid–electrolyte interphase (SEI) cracks.^16,21^ On the other hand, recently Zhang *et al.* were able to produce dendrite free electrodes using FEC/LiNO_3_ electrolyte,^24^ and Mashayek *et al.*^25^ has reported Li-ion diffusion through the SEI of Li-metal batteries. In this work we perform MD simulations of a Li-metal anode covered by a SEI of LiF that is initially cracked, and we analyze the effects of the crack on dendrite formation at few conditions of temperature and C-rate as well as of charging protocols. We choose LiF as SEI because the large amount of theoretical and experimental studies since it is one of the main components of SEI when PF_6_^−^ is used as counterion.^24^ It was also reported that the lithium ions can diffuse through LiF,^25^ which certainly hinders to some extent the entry of lithium ions into the anode but does not completely block their entry.

## Methodology

All molecular dynamics (MD) simulations are performed using the Large-Scale Atomic/Molecular Massively Parallel Simulator (LAMMPS) program developed by Plimpton *et al.*^26–28^ The initial simulation box contains a Li-metal anode covered with a SEI already cracked and an electrolyte of ethylene carbonate (EC) with a 1 M concentration of LiPF_6_. We choose EC as solvent because it is the most popular solvent for current LIBs;^29,30^ one of the reasons is its large dielectric constant of 90.5.^31,32^ The initial simulation box size is 40.8 Å × 54.0 Å × 40.8 Å and contains 640 LiF pairs, 936 Li-metal atoms, 424 EC molecules and 28 LiPF_6_ ion pairs. The initial geometry of the SEI is taken from an earlier work,^33^ which was focused in a LiSi anode. Then for this work, we develop an amorphous Li-metal anode which fits geometrically with the SEI. This yields an amorphous geometry in and around the SEI. The metal Li and SEI has a volume of 29 587 Å^3^, and the electrolyte (EC and LiPF_6_) has a volume of 51 980 Å^3^, corresponding to a density of 1.313 g cm^−3^ of the EC and 1.460 g cm^−3^ of the electrolyte mix. The initial atomic coordinates of the EC molecules and PF_6_^−^ ions are obtained through *ab initio* optimizations of the geometry using Gaussian 09.^34^ These geometries are used to obtain the whole electrolyte using the Packing Optimization for Molecular Dynamics Simulations (PACKMOL).^35^ Snapshots of the simulation box are obtained using the Visual Molecular Dynamics software (VMD)^36^ and 3D visualization Open Visualization Tool (OVITO).^37^

**Fig. 1:**
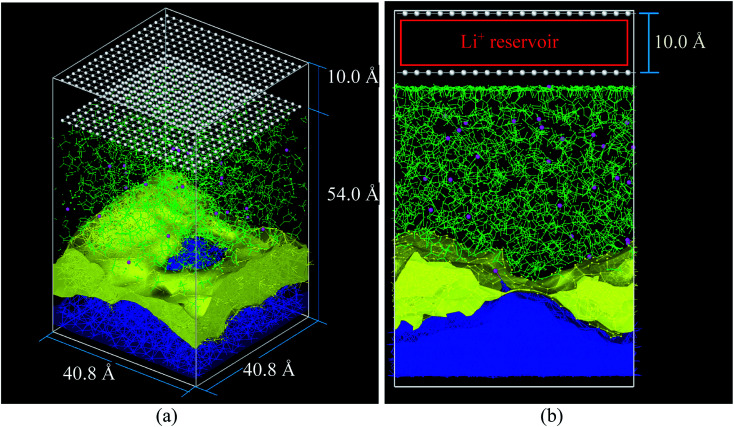
(a) Initial simulation box before the equilibrium, (b) simulation box at the beginning of lithiation. Li-metal (blue), Li^+^ (pink), EC and PF_6_^−^ (green), pseudo atoms (white), LiF (yellow) structure taken from an earlier work.^33^

The cathode in this nanobattery is simplified to a controllable source of ions composed of two layers of 400 frozen strongly bonded pseudo atoms separated by 10 Å (Fig. 1a and b). Li-ions are created in the empty space between the two layers of pseudo atoms. These layers also avoid the periodicity along the electric field direction during all MD simulations (equilibrations and lithiations) which are performed under an NPT ensemble. The nearest neighbor distance between pseudo atoms is set to 2 Å and with a repulsive Lennard-Jones barrier (*ε* = 1.5 kcal mol^−1^, *σ* = 2.5 Å) to avoid interactions between the top and bottom of the simulation box; these barriers prevent any atom traveling from end to end, but they are sufficiently shallow to avoid bond formation, deforming the nearby structures. The layer of pseudo atoms near the electrolyte is at a distance of 2.5 Å from the nearest atoms in the electrolyte. The coordinates of Li-ions created in the Li^+^ reservoir are randomly determined (Fig. 1b).

### Force fields

The interactions between Li-metal atoms and Li-metal atoms with Li^+^ ions are modeled by a second nearest-neighbor (2NN) embedded MEAM force field,^38,39^ as it was used in a previous work.^40^ The MEAM interaction is given by the equation:
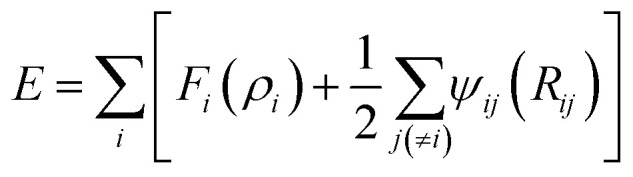
where *F*_*i*_(*ρ*_*i*_) is the embedding function, *ρ*_*i*_ is the background electron density at the site atom *i* occupies, and *ψ*_*ij*_(*R*_*ij*_) is the pair interaction between atoms *i* and *j* at a distance *R*_*ij*_. The background electron density *ρ* is composed of several partial electron density terms. Each partial electron density is a function of atomic configuration and atomic electron density. The quantities used to generate the functions *F*_*i*_ using density functional theory are given in Table 1. Since Li^+^ can be seen as a hole (e^+^ charge) plus a neutral Li^0^ as per coulombic interactions only, Li^+^–Li can be partitioned into a Li–Li interaction plus a e^+^–Li^0^ interaction. The latter reduces to zero and the former is only useful for distances smaller than 2.5 Å due to the fact that Li^+^ is reduced at distances smaller than 2.5 Å from the Li-metal atoms. Therefore, we use the same MEAM force field parameters of Li–Li interactions for the interaction between both Li-metal and Li^+^ at distances smaller than 2.5 Å.

**Table 1:** MEAM potential parameters for Li and Li^+^^38^

Parameter	Value
Lattice type	BCC
*E*_c_ (eV)	38.05
Lattice constant (Å)	3.509
*Z*	8
Weight (g mol^−1^)	6.939
*r*_c_ (Å)	10

*E*_c_ is the energy per atom, *z* is the number of nearest neighbors in the reference structure and *r*_c_ is the cut off distance. The equilibrium distance in a lithium diatomic molecule using a MEAM force field is 2.419 Å,^40^ which is close to the experimental distance of 2.672 Å.^41^

The interactions among the SEI (LiF) atoms are modeled with the Born–Mayer potential,^42^
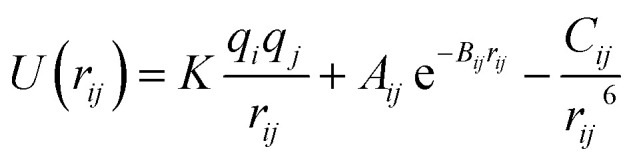
where *r*_*ij*_ is the distance between ions *i* and *j*, *q*_*i*_ and *q*_*j*_ are the charge of each ion, *A*_*ij*_, *B*_*ij*_ and *C*_*ij*_ are parameters defined for each pair of atoms (Table 2); these parameters are taken from previous work.^42,43^

**Table 2:** Born–Mayer potentials parameters for LiF^42^

Pair	*A* (J mol^−1^)	*B* (Å^−1^)	*C* (J Å^6^ mol^−1^)
Li–Li	1.03	1.442	10.0
Li–F	0.28	2.934	−4.0
F–F	1.31	3.695	13.9

All the interaction between Li^+^, P, F, C, O, H atoms (electrolyte atoms) are modeled with an updated ReaxFF developed by Mahbubul *et al.*^44^ The ReaxFF is a bond-order based potential, including a polarizable charge calculation^45–47^ that allows the breaking and formation of new bonds and, in consequence, the formation or dissociation of molecules during the simulation.

Nonbonded interactions between the solvent and Li-metal, solvent and SEI, SEI and Li-metal are simulated using a Lennard-Jones (L-J) potential in conjunction with coulombic parameters (Table 3).

**Table 3:** Nonbonded Lennard-Jones and coulombic parameters

Atom	*ε* (kcal mol^−1^)^48,49^	*σ* (Å)^48,49^	*q* (e)^49^
F^−^	0.028	2.934	−0.34
P	0.131	3.695	1.07
PF_6_^−^			−1.00
Li^+^	0.103	1.442	1.00
F^−^	0.028	2.934	−1.00
LiF			0.00
O=	0.210	2.960	−0.65
O–	0.170	3.000	−0.47
C(sp^3^)	0.105	3.750	1.10
C(sp^2^)	0.066	3.500	0.03
H	0.030	2.500	0.10
Pseudo atom	1.500	2.500	0.00

It is important to consider that the simulation times used in this work and the experimental times in commercial batteries cannot be compared directly. The simulation time and experimental times are calculated and measured in different scales; thus, we can accelerate the lithiation process and perform simulations in a reasonable computational time. We use large electric fields, which are explained in the Results section. The lithiation process in these simulations only takes a few ps instead of minutes or hours as it would in a real battery.

We define three protocols to charge the battery:

(1) Pulse train (PT): a pulse of 10 Li^+^ is created at random locations in the Li^+^ reservoir every 4 ps, regardless of the number of Li^+^ in the box or the number of Li^+^ that are reduced at the anode. Thus, the net charge is not necessarily 0 and current is roughly constant.

(2) Constant number of ions (CI): a Li^+^ is created at random location in the Li^+^ reservoir every moment that a Li^+^ is reduced at the anode, *i.e.*, concerted redox reactions. Thus, the number of Li^+^ in the box remains constant and the total charge is always 0. Under this protocol, current increases with time because Li^+^ travel distance to reach the anode becomes shorter and shorter due to plating; therefore, Li^+^ ions reach the anode and new Li^+^ ions are created more frequently. By comparison, other protocols keep Li^+^ creation rate constant, and the Li^+^ travel distance does not change the current.

(3) Constant current (CC): a Li^+^ is created at random location in the Li^+^ reservoir every 0.4 ps. The current is closer to be constant than in the PT protocol and the net charge on the whole battery is not necessarily 0 (there must be 28 Li^+^ in the electrolyte to have 0 total charge).

In these three protocols, when a Li^+^ reaches the anode, it transforms to a Li-metal atom. We consider a Li^+^ as having reached the anode when it is at a distance smaller than 2.5 Å from any Li-metal atom.

### Lithiation simulations

We consider eight cases, using several electric fields, C-rates, temperatures and protocols to determine what factors favor or restrict lithium dendrite formation. We also use different values of electric field for the Li^+^, Li, F, and the other atoms in the box. We apply strong electric fields between 1.46 and 1.5 V Å^−1^ to Li^+^ to accelerate the lithiation process.

The molecular dynamics of all cases are performed under a NPT ensemble with the temperature set at 300 K, but due to the arrival of new ions to the simulation box and the chemical reactions in the electrolyte, the temperature raises to more than 300 K between 325 K and 410 K in average depending of each case. These data are provided and analyzed in the result part, thus short temperature relaxation times of 30 and 50 fs (60 and 100 timesteps) are used to avoid high temperatures that could melt lithium. Also, 100 timesteps is a reasonable value for the temperature relaxation in MD simulations.^50^ For pressure relaxation at 1 atm we use 10 ps, which is also in the order of the reasonable value by the authors of the program and other MD specialists,^51^ 0.5 ps (1000 timesteps). This relatively large pressure relaxation-time does not allow large changes of volume, which changes due to new few hundreds Li when the total sample has 7880 atoms. Therefore, volume changes are very small. The low change of volume during the whole simulation allows us to use a large pressure relaxation time of 10 ps, which is a time comparable with the times simulated under electrical field. The main parameters of the 8 cases considered are summarized in Table 4.

**Table 4:** Input parameters for each case where 
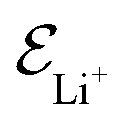
 is the electric field for Li^+^, 
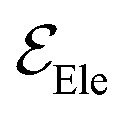
 is the electric field apply for EC and PF_6_^−^, *t*_Trel_ is the temperature relaxation time and *Q*_Eq_ indicate whether charge equilibration is used or not

#	Case name	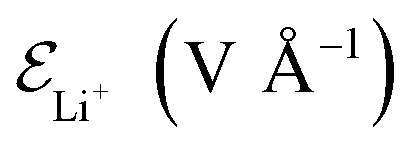	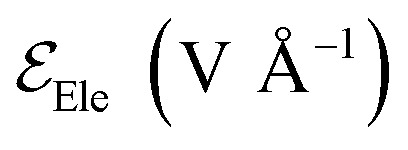	Protocol	*t*_Trel_ (fs)	*Q*_Eq_
1	*ht*_Trel_–PT	1.5	0.5	PT	50	No
2	*ht*_Trel_–CI	1.5	0.5	CI	50	No
3	*mt*_Trel_–CI	1.47	0.5	CI	40	No
4	*lt*_Trel_–CI	1.46	0.5	CI	30	No
5	Std	1.5	0.5	CC	50	No
6	*Q*_Eq_–PT	1.5	0.0	PT	50	Yes
7	*Q*_Eq_–CI	1.5	0.0	CI	50	Yes
8	*Q*_Eq_–CC	1.5	0.0	CC	50	Yes

Electric fields are applied in different ways to atoms and molecules as explained next. We do not apply electric fields to the LiF because an electric field of 1.5 V Å^−1^ can break the LiF material, mainly because of the large and opposite ionic charges of ±1. The coulombic attraction force and dissociation force by an electric field of 1.5 V Å^−1^ are 1.04 nN and 2.4 nN, respectively, in a crystalline structure. The LiF SEI is already cracked with an amorphous structure and several broken bonds, and its structure dissociates easily. We apply an electric field of 0.5 V Å^−1^ to the EC and PF_6_^−^ in cases 1–5, which do not have charge equilibration (no *Q*_Eq_) because a stronger field (*e.g.*, of 1.5 V Å^−1^) only increases their mobility, making the lithiation slower. Cases 6–8 have charge equilibration (using *Q*_Eq_) on the atoms that compose the electrolyte, thus the electrolyte atom charges can change. This characteristic helps reactions to take place and makes a more realistic simulation. A problem with performing a charge equilibration is that the computational time increases due to two reasons: the necessary additional calculations for *Q*_Eq_ to get the charge of each atom, and the charge change of a Li^+^ from +1 to a value around +0.55, which means the electric field applies less force to the ions. We ran the previous simulation, and based in the observed results, we estimate that would be approximately 11 times longer than cases without *Q*_Eq_; therefore, to avoid this problem in cases 6–8 (using *Q*_Eq_), the electric field applied to EC and PF_6_^−^ is 0. This change makes a faster lithiation possible and uses less computational resources because without electric field, the EC and PF_6_^−^ vibrate less and hinder the passage of the ions less.

Case 5 is the standard or reference one due to the fact that is one of the most used methods to charge a battery. Thus, extended calculations such as the compressive stress and porosity are performed for this case.

## Results and Discussions

**Fig. 2:**
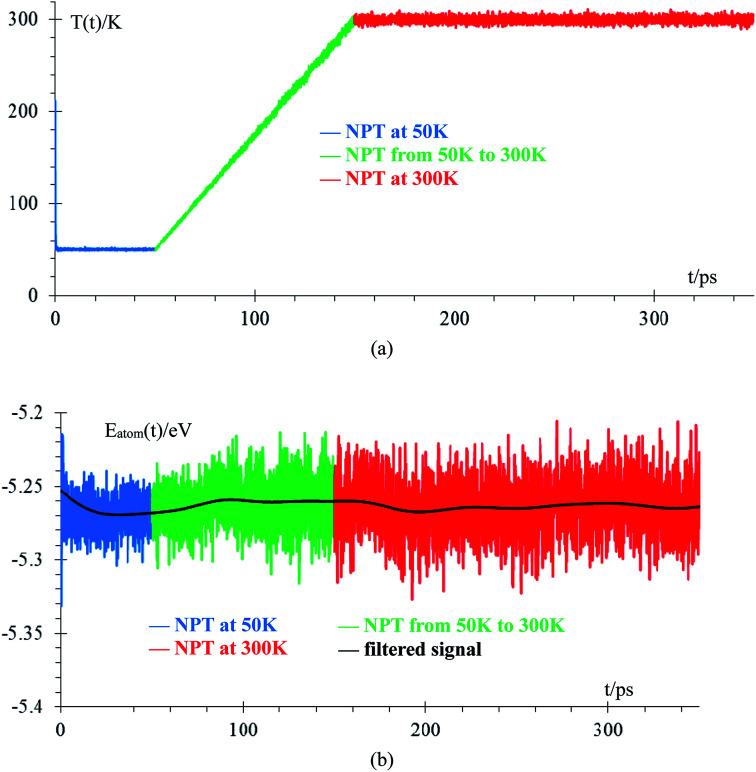
Time evolution of equilibration runs: (a) temperature, (b) average energy per atom (∼7000 atoms). Filtered signal is obtained by applying a discrete time average function, *E*_*n*_(*t*) = 1/4*E*_*n*−1_(*t* − 1) + 1/2*E*_*n*−1_(*t*) + 1/4*E*_*n*+1_(*t* + 1), where *E*_0_ is the original signal and *E*_10000_ is the filtered signal, with time intervals between samples of 50 fs (100 steps). This averaging is used in all filtered signals, unless stated otherwise.

We performed an equilibration of the box in three stages: the first stage is performed at 50 K for 50 ps to eliminate any hot spots in the initial geometry. Then, in the second stage, the temperature is increased from 50 K to 300 K at a rate of 2.5 K ps^−1^, and finally, in the third stage, the box is equilibrated at 300 K for 200 ps. These 3 stages are performed using the NPT ensemble with a temperature relaxation time of 50 fs and 1 atmosphere, with a pressure relaxation time of 10 ps. Temperature noise increases as temperature increases until reaching 300 K ± 10 K (3% error margin) and we consider the sample equilibrated (Fig. 2a). The total energy per atom for the three stages of the equilibration are shown in Fig. 2b. The filtered average energy for all atom signals is also shown. Increasing the temperature from 50 K to 300 K minimally changes the average energy, less than 1%.

**Fig. 3:**
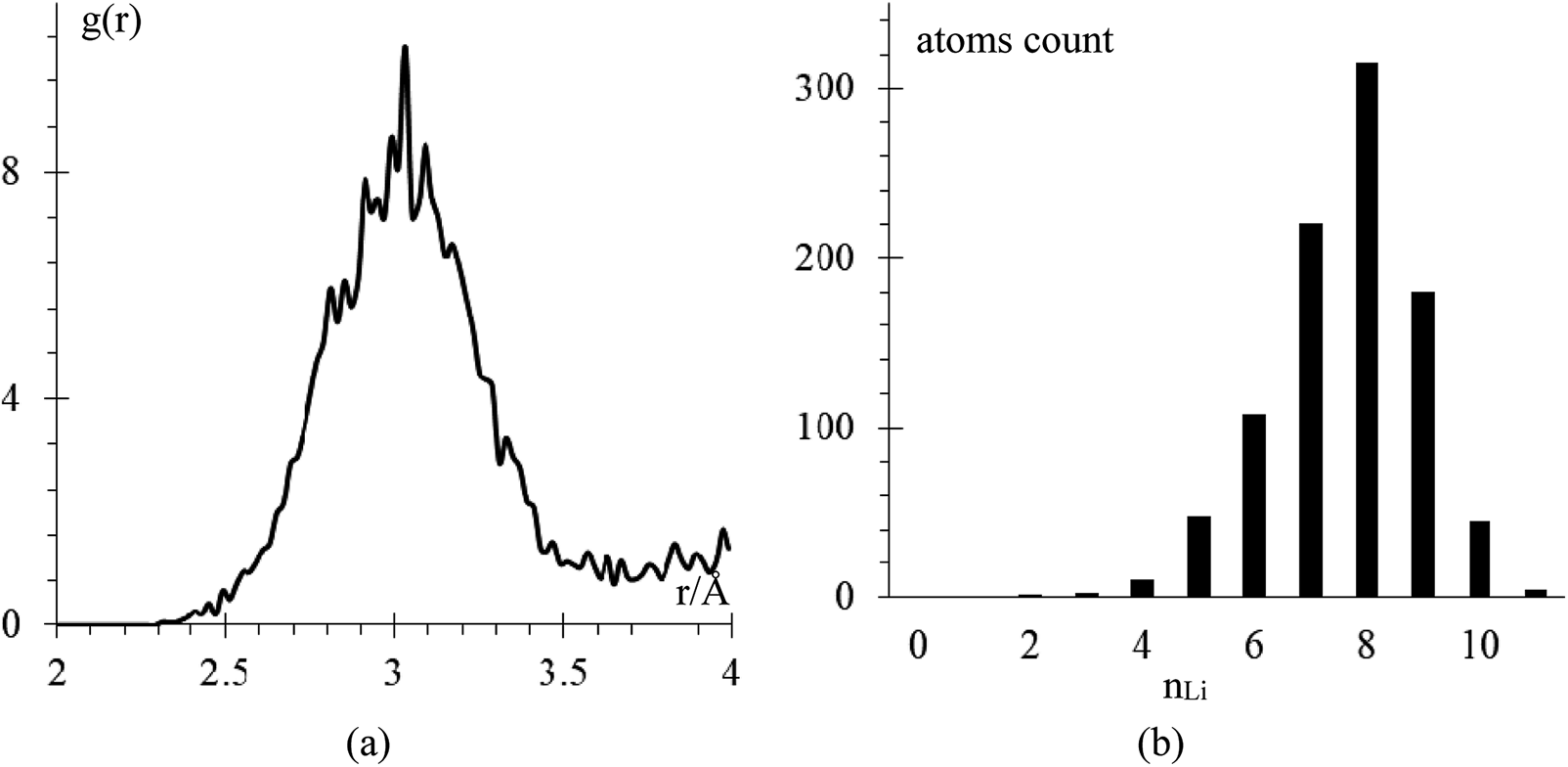
(a) RDF Li–Li after equilibration (b) number of Li atoms in the anode having *n*_Li_ atom neighbors *versus* number *n*_Li_. There is a total of 936 Li-atoms in the anode.

The amorphous Li-metal anode stays amorphous after equilibration, but it keeps the properties of bulk Li-metal; for example, the anode has a volume of 18 618 Å^3^ and a density of 0.579 g cm^−3^, which is close to the experimental value of 0.59 g cm^−3^.^6^ Nearest neighbor distance in the anode is 3.038 Å (Fig. 3a), and the BCC structure has 8 nearest neighbors, which is the most common nearest neighbor number in this anode by the end of equilibration (Fig. 3b). To determine the number of nearest neighbors, we define the Li–Li cutoff distance as 3.273 Å, taken from the average of the experimental nearest neighbor distance in Li-metal of 3.038 Å and the second-nearest neighbor distance of 3.508 Å.

**Fig. 4:**
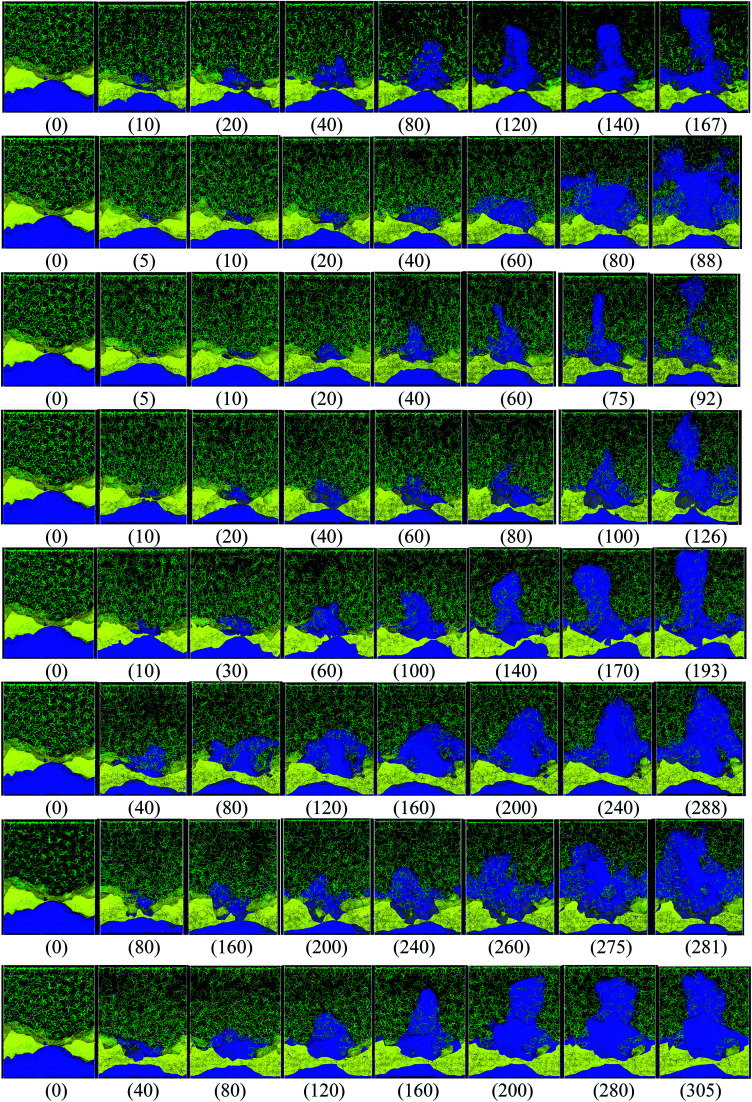
Dendrite growth for cases 1–8, respectively (shown from top to bottom rows, in the same order as shown in Table 4), the label (inside parentheses) indicates the time in ps when the snapshot is taken. Color code: Li-metal (blue), Li^+^ (pink, very hard to see them), EC and PF_6_ (green), LiF (yellow).

Different protocols yield different short-circuit times. We define the short-circuit time as the time that takes to the dendrite to growth and touch the cathode as shown in Fig. 4. The charge equilibration roughly doubles the short-circuit time as it reduces the charge of any Li^+^, thus increasing the short-circuit time. Therefore, we classify the 8 cases in Table 4 into two groups. Cases 1–5 do not perform charge equilibration (no *Q*_Eq_), and cases 6–8 do (*Q*_Eq_). A lithium dendrite forms similarly in 7 of the 8 cases. The exception is the case of *ht*_Trel_–CI (case 2), where the lithium dendrite grows in a very irregular pattern (Fig. 4).

**Fig. 5:**
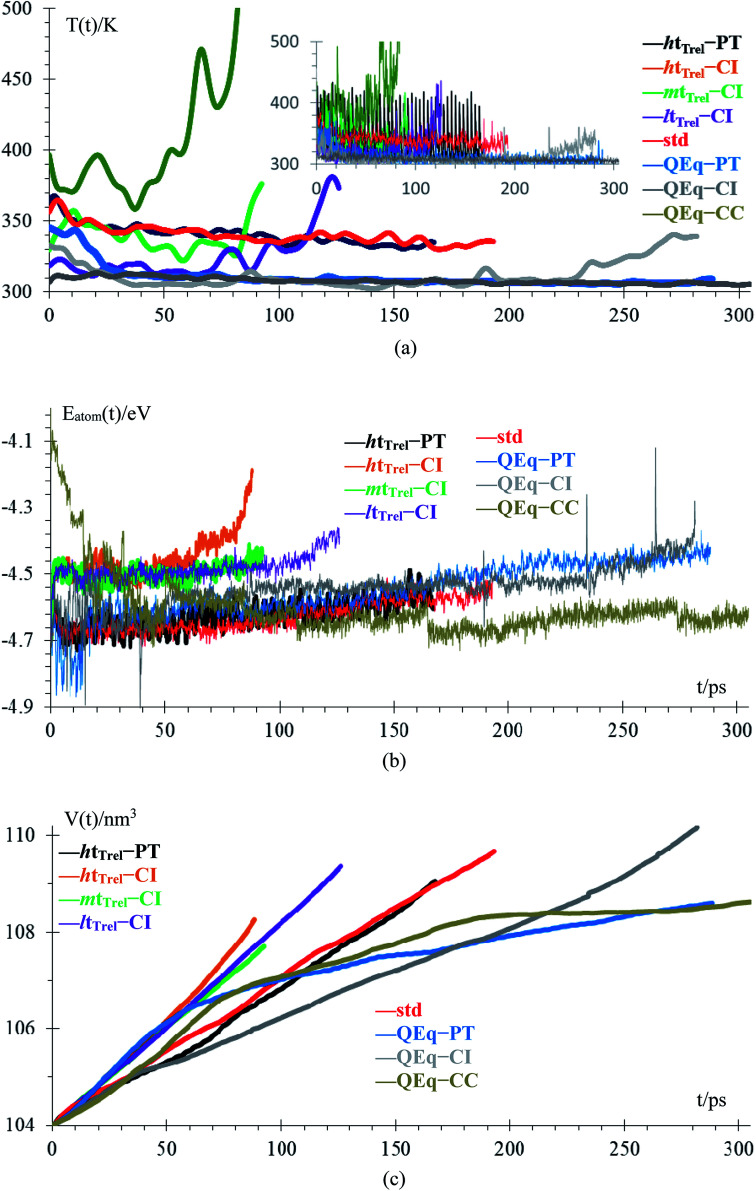
(a) Low-pass filtered temperature signal; original signal in the inset, (b) energy per atom, (c) volume of the box.

Temperature and energy per atom change very differently. Temperature (Fig. 5a) is always around 300 K for cases 6–8, *i.e.*, they have similar average temperatures; therefore, these cases could dictate the protocol for better charging of the battery. For the case of the pulse train, every 4 ps, there is a peak in the temperature every 4 ps. When 10 new Li^+^ enter to the electrolyte, they produce a strong disturbance to the box, which increases random motion and therefore, the temperature increases. Total energies per atom for each case are shown in Fig. 5b. Except for the case 2, all other seems to equilibrate to an steady state; however the strong fluctuations due to the metal–electrolyte interactions can be clearly observed. Volume variations are between 3% and 6%, and variation in length of the simulation box are between 1% and 2% (Fig. 5c). Thus the maximum difference of final volumes is less than 3%, which means that the maximum different in final heights will be less than 1%; therefore, we can assume for the sake of comparisons that all cases yield the same final height at the end of the simulations.

**Fig. 6:**
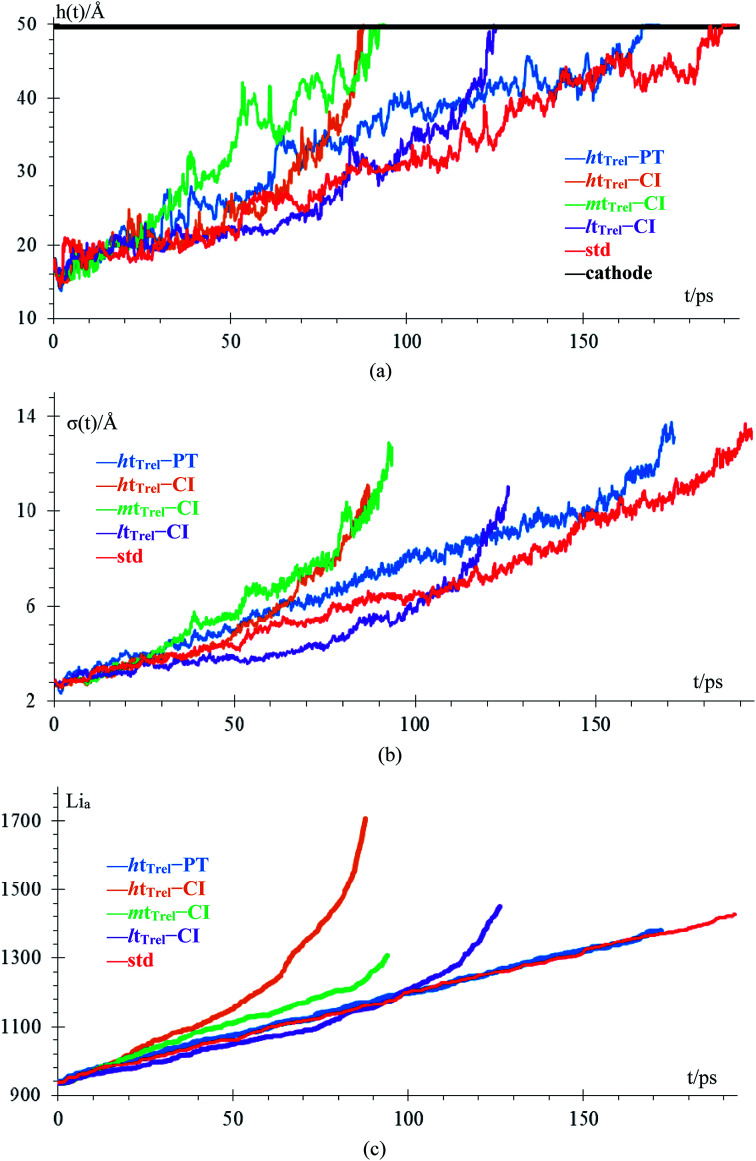
Time evolution of (a) height of the highest dendrite peak, (b) standard deviation of heights of lithium atoms in the anode surface representing the dendrite formation, (c) number of Li atoms in the anode (Li_a_).

**Fig. 7:**
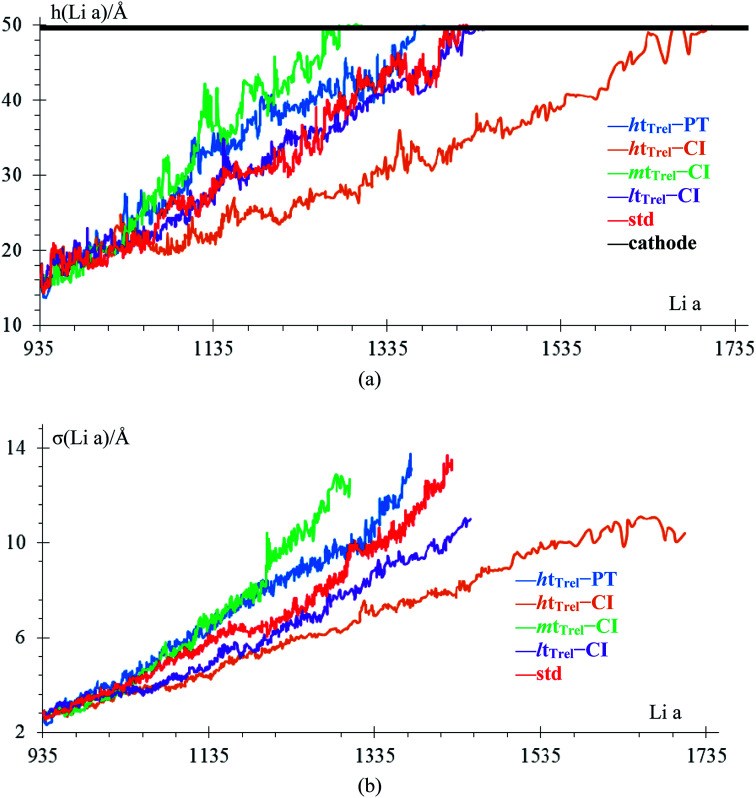
(a) Height, *h*, of the highest dendrite peak *vs.* number of lithium atoms in the anode (Li_a_), (b) standard deviation of the heights of lithium atoms in the anode surface, *σ*, of the dendrite formation. Standard deviation of heights of lithium atoms in the anode surface increase means that the surface of the lithium dendrite does not grow uniformly (spatially).

We compare first the cases 1–5 (no *Q*_Eq_) to have a better idea of how the dendrite grows in each case. We calculate the height of the highest dendrite peak corresponding to the highest Li atom that belongs to the dendrite (Fig. 6a). We calculate the standard deviation of heights of lithium atoms in the anode surface with respect to the anode surface (Fig. 6b) to analyze how the shape of the dendrite evolves due to the arrival of lithium atoms in each case. The dendrite height always increases (spatially), and the standard deviation of the heights of lithium atoms in the anode surface increases with time; therefore, the growth is never uniform. We calculate the number of lithium atoms in the anode in each case (Fig. 6c), which is equivalent to the stored potential energy in the battery. The number of lithium atoms in the anode before the short-circuit is different in each case; thus, each case can store a different amount of energy before the short-circuit. The standard deviation of the heights of lithium atoms in the anode surface is given by,
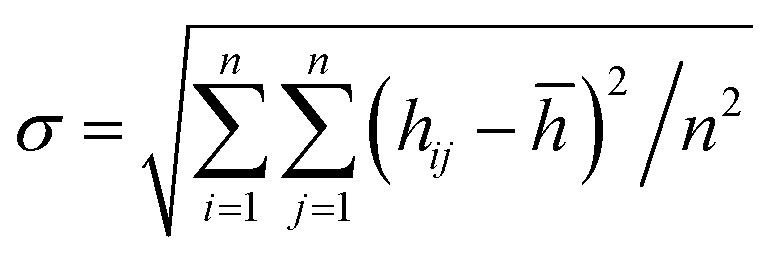
the plane *x*–*y* of the cell is divided in *n* × *n* (*n* = 13) equal square sectors, thus, each square sector has a side length of ∼3.14 Å, which is larger than the Li-metal bond distance of 3.04 Å.^52^ Thus, at least one Li-metal atom is on the surface of each sector. *h*_*ij*_ is the height of the highest Li-metal in the sector *ij*, and *h̄* is the average height among the highest Li-metal atoms from each sector,
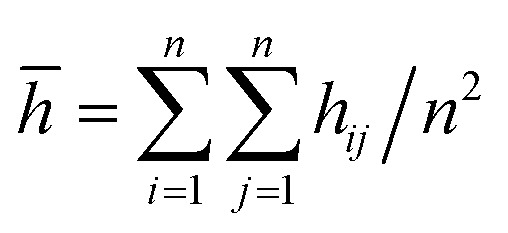


Although we compare cases 1–5 (no *Q*_Eq_), the time comparisons are not a good way to compare them because of the different total simulation times. A better way to compare the cases 1–5 is by using the stored energy, which is equivalent to the number of lithium atoms in the anode (Fig. 7). Height of the highest dendrite peak and standard deviation of heights of lithium atoms in the anode surface tend to grow linearly respect to number of lithium atoms in the anode (stored energy) in all cases.

In cases 1–5, lithium concentrates on the LiF crack, starting the formation of a dendrite. Except for *ht*_Trel_–CI, the dendrite becomes taller and sharper; however, for *ht*_Trel_–CI, the dendrite grows very differently than in the other cases. The speed of growth of the dendrite is different for each case, determining the possible rate of charge for a safe use of the battery. We calculate the average temperature during each lithiation and the Li-metal amount before the short circuit in cases 1–5, or no *Q*_Eq_ cases (Table 5). Notice that the average temperatures in all cases is always above 325 K, which is higher than the melting temperature of EC (310 K).

**Table 5:** Comparison of average temperatures and total lithiation times for cases 1–5 (no *Q*_Eq_)

#	Case	Li count	*t*_total_ (ps)	*T*_ave_ (ps)
1	*ht*_Trel_–PT	1368	166.8	340.5
2	*ht*_Trel_–CI	1678	88.4	410.7
3	*mt*_Trel_–CI	1275	92.5	339.4
4	*lt*_Trel_–CI	1402	126.2	325.0
5	Std	1408	193.2	340.3

In the case of *ht*_Trel_–CI, the battery can be charged more than in the other cases before the dendrite reaches the top of the electrolyte. This is because the temperatures are higher than in any of the other cases. The velocity of all the atoms increases, and dendrite grows in all directions, making a dendrite with high volume but low height. Therefore, a high temperature could hinder dendrite formation, and the low temperature helps the lithium dendrite growth. The previous result is in accord with previous experiments^53,54^ and previous computational works.^55^

In the case of *ht*_Trel_–CI, 742 Li-ions reach the anode in 88 ps while in the case of *mt*_Trel_–CI, 303 Li-ions reach the anode in 88 ps. In the case of *lt*_Trel_–CI in the same time period, 217 Li atoms reach the anode. Using this data, we calculate the C-rate for cases 2–4 (Table 6). Assuming the battery has a capacity of 55 A h, the C-rates are scaled to a realistic value, comparing the size of the battery with a typical 55 A h lithium ion battery using the following relationship:
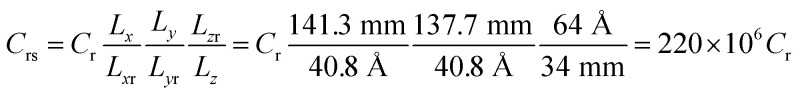
where *C*_rs_ is the scaled to C-rate, *C*_r_ is C-rate, *L*_*x*_, *L*_*y*_, *L*_*z*_ are the dimensions of the nanobattery, and *L*_*x*r_, *L*_*y*r_, *L*_*z*r_ are the dimension of the lithium ion cell model LP 32770, yielding a scale factor of 220 M. We scale the C-rate and dendrite growth rate to have a relatively good idea of how our results from nanobatteries would scale to be useful in the design of realistic size batteries. It is now a very primitive and crude scaling procedure that may eventually evolve and make our atomistic nanobattery calculations able to deal with realistic macroscopic sizes.

**Table 6:** Equivalent C-rates used in cases 2–4. *n*_Li_ = number of Li-ions reaching the anode, *I* = total current of Li^+^ in the battery, *t*_1_ = time to charge 55 A h, C-rate in our battery (*C*_r_) is *C*_r_ = 1 h/*t*_1_, *C*_rs_ = 220 × 10^6^*C*_r_. C-rate analysis performed in a snapshot taken 88 ps right after the lithiation onset

Case	*n*_Li_	*I* (A g^−1^)	*I* (μA)	*t*_1_ (h)	*C*_r_ (μC)	*C*_rs_ (C)
*ht*_Trel_–CI	742	13 904	1.349	40, 770, 941	0.0245	5.390
*mt*_Trel_–CI	303	13 883	0.551	99, 848, 511	0.0100	2.200
*lt*_Trel_–CI	217	13 897	0.395	139, 240, 506	0.0072	1.584

The case of *lt*_Trel_–CI can be charged more than the *mt*_Trel_–CI, therefore, a high C-rate helps dendrite growth; this result is in agreement with previous experiments.^56,57^ High C-rate is less important than high temperature for the grow of dendrites. Because of this, the case at *ht*_Trel_–CI can support more lithium atoms in its anode than any other case.

Due to the fact that an electric field increases the velocity of Li^+^ ions, lithium dendrite has a high growth rate between 17 and 40 m s^−1^. Therefore, to compare to experimental results, we need to scale the growth rate using the same factor that we used previously to scale the C-rate (Table 7).

**Table 7:** Scaled C-rates and scaled dendrite growth rates compared with experimental results, *G*_r_ = (*h*_max_ − *h*_min_)/*t*_short-circuit_, where *G*_r_ = growth rate; *h*_max_ and *h*_min_ are the maximum and minimum heights (Fig. 6a and 7a), and *G*_rs_ = *G*_r_/(220 × 10^6^) is the estimated scaled growth rate

Case	*T*_ave_ (K)	*C*_rs_ (C)	*I* (μA)	*G*_r_ (m s^−1^)	*G*_rs_ (mm h^−1^)
*ht*_Trel_–PT	340.5	1.604	0.40	20.71	0.339
*ht*_Trel_–CI	410.7	5.390	1.39	39.28	0.643
Std	340.3	1.604	0.40	17.47	0.286
Experiment 1 (ref. 58)	293	0.481	1000		0.92
Experiment 2 (ref. 58)	293	0.675	1000		1.70
Experiment 3 (ref. 58)	293	2.000	1000		2.18

Scaled growth rate is in the same order of magnitude than observed in experiments.^58^ Actual values of the simulated scaled growth rates are a little lower than in the experiments because temperatures in our simulations are higher than 300 K, which deter dendrite formation.

**Fig. 8:**
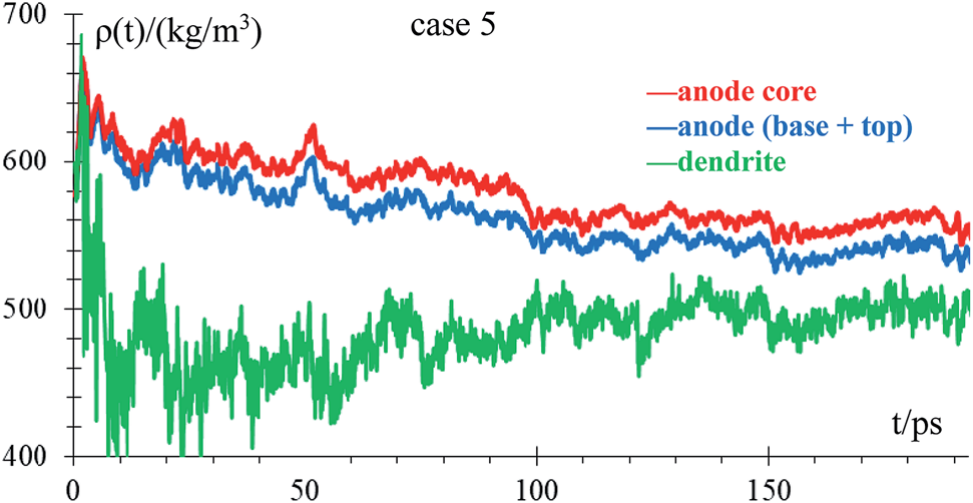
Time evolution of the anode core density, dendrite, and whole anode.

We analyze how the time evolution of the anode density changes during lithiation and whether the density in the anode core is different from that of the dendrite in the standard case. We consider a Li-metal atom to be part of the top of the anode if its height is greater than the height of highest Li-metal at time 0. In case the Li-metal atoms are not part of the top, we consider them part of the anode core regardless of their neighbors. The dendrite has very few atoms at the beginning of lithiation (10 atoms), and the graph of the density of this part has a lot of noise during the firsts ps. Density decreases with time from the beginning of the lithiation (theoretical density is 579 kg m^−3^) due to the anode becoming porous, with the most porous part being the dendrite. This porosity allows the formation of a low-density lithium dendrite (Fig. 8).

**Fig. 9:**
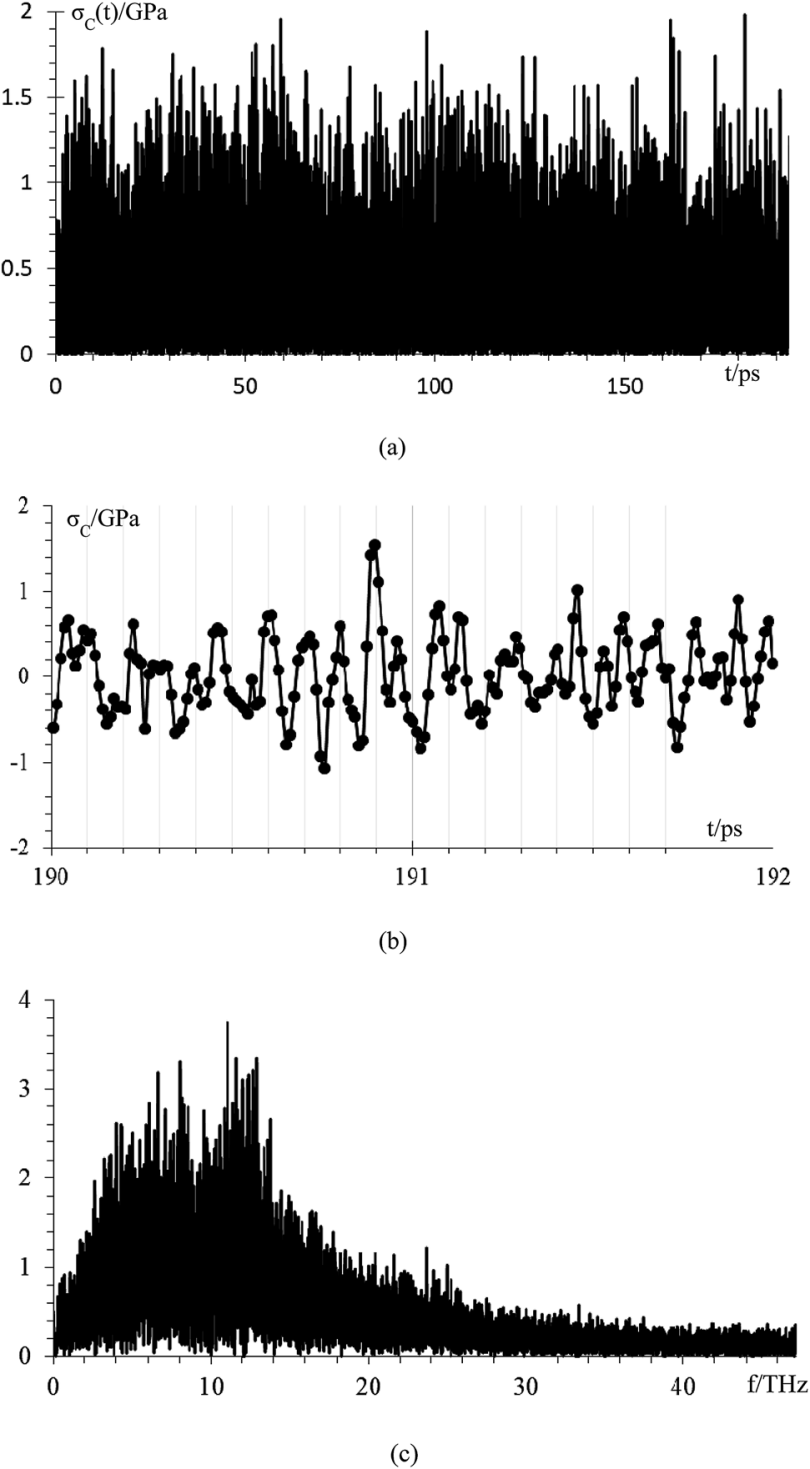
(a) Compressive stress from Li-metal to electrolyte and SEI for case 5, (b) compressive stress from 190 ps to 192 ps, (c) frequency spectrum of the compressive stress signal.

We analyze the compressive stress that the Li-metal anode applies to electrolyte and SEI in standard case (Fig. 9), which performs at an average temperature of 340.3 K, and we calculate the compressive stress from
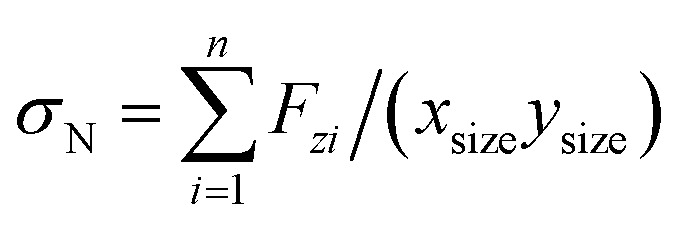
where *n* is the number of Li-metal atoms in the anode, *F*_*zi*_ is the force applied to the lithium *i* by all other atoms in the cell in the *z*-direction, *x*_size_ and *y*_size_ is the box size in the *x* and *y* axis respectively, and *x*_size_*y*_size_ is equivalent to the transversal area due to the dendrite growth in *z*-direction. The previous summation could be positive or negative, but we only consider the positive results (Fig. 9a) because a negative result means that the compressive stress is applied to the layer of pseudo atoms instead of the SEI.

Compressive stress could reach peaks between 1.5 GPa and 2 GPa (Fig. 9a); therefore, the SEI must support this amount of compressive stress to avoid the lithium dendrite formation in a Li-metal anode without structural damage. We estimate, based on the data points of a final part of the signal from 190 to 192 ps (Fig. 9b), that the principal frequency of compressive stress signal is approximately 11 THz, and our sampling frequency is approximately 100 THz which corresponds to taking one sample every ∼9 fs. Therefore, our sampling frequency follows quite well the shape of the signal. To confirm that our sample frequency is appropriate, we calculate the frequency spectrum of the compressive stress signal (Fig. 9c). The principal frequencies are below 15 THz and after 40 THz there are only noise as we are able to remake the original signal from the frequency spectrum up to 40 THz yielding an error margin of less than 1%; therefore, we can consider 40 THz as the maximum frequency and as the sampling frequency of 100 THz is more than twice the maximum frequency of compressive stress, and according to Nyquist–Shannon sampling theorem,^59^ our sampling frequency is appropriate to represent in the frequency domain, the original time-domain signal.

**Fig. 10:**
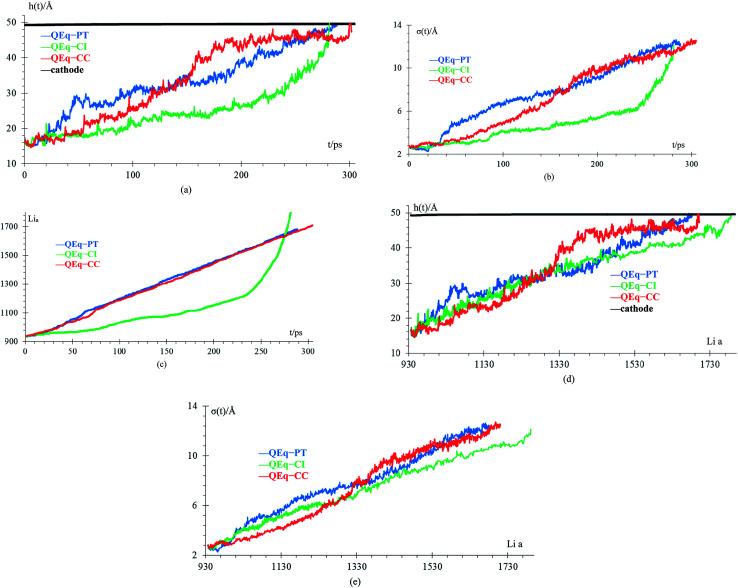
(a) Height of the highest dendrite peak *vs.* time, (b) standard deviation of heights of lithium atoms in the anode surface *vs.* time, (c) number of lithium atoms in the anode (Li_a_) *vs.* time, (d) height of the highest dendrite peak *vs.* number of lithium atoms in the anode (Li_a_), (e) standard deviation of heights of lithium atoms in the anode surface *vs.* number of lithium atoms in the anode (Li_a_).

We compare cases 6–8 using *Q*_Eq_ (Fig. 10), and we get that the amount of Li-metal before the short circuit at constant current is greater than at the pulse train, indicating that charging the battery at constant current is more effective than using a pulse train. In each case, a lithium dendrite grows. Li fills in the crack, even having a uniform lithiation, then forms a dendrite until the short-circuit takes place. The standard deviation of heights of lithium atoms in the anode surface also increases with time. All the behavior described before is the same as cases 1–5 (no *Q*_Eq_), which is not being changed by the electronic distribution. Consequentially, all results that we get for cases without *Q*_Eq_ would be similar if we made them with charge equilibration.

Finally, case 5 (std) and case 8 (*Q*_Eq_–CC) have the same lithiation rate of 1 Li^+^ every 0.4 ps, but the case *Q*_Eq_–CC can be charged more before the dendrite reaches the cathode. The main difference between these 2 cases is the use of charge equilibration, which facilitates the reaction in the electrolyte delaying the formation of lithium dendrites.

## Conclusions

It is found that the electrolyte reactions delay the dendrite formation. Previous works showed that parts of the lithium dendrite break and remain in the electrolyte through charge/discharge cycles resulting in the formation of dead Li.^60^ In this work, we show that the dendrite is considerably more porous than the rest of the anode, allowing the formation of larger dendrites with low density and vulnerability to breaking, forming dead Li over multiple charge/discharge cycles. Also, the dendrite never grows spatially homogeneous even under uniform lithiation, but lithium always has a preference to deposit at specific parts of the anode, which causes the shape of the dendrite to change vertically with respect to the surface, increasing the distance between the anode peaks and anode valleys. We conclude that the mere presence of a cracked SEI greatly favors the lithium dendrite formation at the crack. Even if lithiation is uniform throughout the area, the lithium metal naturally will accumulate over the crack, forming a dendrite, and this takes place regardless of how the battery is charged. Certainly, LiF crack is the main driver for the growth of the dendrite. However, Li-ions also go through the uncracked areas of the LiF and get reduced and deposited on the metallic Li-anode underneath, which is still covered by the LiF shell. These deposited Li increase the internal pressure toward further expansion and more cracking of the LiF shell and they actually contribute and help the growth of the dendrite from the bottom in addition to the growth from the top caused by those ions falling on top of the crack or of the growing dendrite tip. Finally, we estimated that the electrolyte must support a compressive stress of at least 2 GPa to avoid the dendrite formation without suffering structural damage at 340 K.

## Conflicts of interest

There are no conflicts of interest to declare.

## Supplementary Material
